# A complex DICER1 syndrome phenotype associated with a germline pathogenic variant affecting the RNase IIIa domain of DICER1

**DOI:** 10.1136/jmedgenet-2020-107385

**Published:** 2020-11-18

**Authors:** Emeli Pontén, Sofia Frisk, Fulya Taylan, Raquel Vaz, Sandra Wessman, Leanne de Kock, Niklas Pal, William D Foulkes, Kristina Lagerstedt-Robinson, Ann Nordgren

**Affiliations:** 1 Department of Molecular Medicine and Surgery (MMK), Karolinska Institute, Stockholm, Sweden; 2 Clinical Genetics, Karolinska University Hospital, Stockholm, Sweden; 3 Oncology-Pathology, Karolinska University Hospital, Stockholm, Sweden; 4 Departments of Human Genetics, Oncology, Medicine, McGill University, Montreal, Québec, Canada; 5 Department of Pediatric Oncology, Karolinska University Hospital, Stockholm, Sweden

**Keywords:** genetic predisposition to disease, genetics, medical, genomics

## Abstract

**Background:**

Germline pathogenic variants in *DICER1* cause DICER1 syndrome, an autosomal dominant, pleiotropic tumour predisposition syndrome with variable expressivity and reduced penetrance for specific dysplastic and neoplastic lesions. Recently, a syndrome with the acronym GLOW (*G*lobal developmental delay, *L*ung cysts, *O*vergrowth, *W*ilms tumour) was described in two children with mosaic missense mutations in hotspot residues of the DICER1 RNase IIIb domain.

**Methods:**

Whole genome sequencing, exome sequencing, Sanger sequencing, digital PCR and a review of Wilms tumours with *DICER1* RNase III domain mutations were performed.

**Results:**

A de novo heterozygous c.4031C>T (p.S1344L) variant in the sequence encoding the RNase IIIa domain of *DICER1* was detected. Clinical investigations revealed a phenotype that resembles the GLOW subphenotype of DICER1 syndrome.

**Conclusion:**

The phenotypic overlap between patients with p.S1344L mutation and GLOW syndrome provide clinical support for recent discoveries that RNase IIIa-Ser1344 site mutations impede miRNA-5p biogenesis analogous to *DICER1* hotspot mutations in the RNase IIIb domain. We show that an individual with a heterozygous germline p.S1344L mutation has a severe form of DICER1 syndrome (‘DICER1 syndrome plus’), with notable features of intellectual disability, macrocephaly, physical abnormalities, Wilms tumour and a well-differentiated fetal adenocarcinoma of the lung.

## Introduction

The *DICER1* (MIM *606241) gene located at 14q32.13 is important for embryogenesis and early somatic development. Residing in the cytoplasm, the DICER1 protein is an endoribonuclease (RNase) III cleaving double-stranded RNA. The enzyme is crucial for producing miRNAs, as it processes precursor strands (pre-miRNA) whereby two single-stranded miRNA molecules are produced, named by their prime end origin (3p/5p miRNA).^1^

DICER1 syndrome (MIM #601200) is an autosomal dominant, pleiotropic tumour predisposition syndrome with variable expression and reduced penetrance of benign and malignant tumours, commonly pleuropulmonary blastoma (PPB), cystic nephroma, Sertoli-Leydig cell tumour and hyperplastic proliferations such as multinodular goitre. The most frequently observed mechanism underlying tumour formation in DICER1 syndrome is the presence of biallelic alterations that include germline loss of function (LOF) pathogenic variants, combined with somatic in trans mutations in the sequence encoding the hotspot residues of the RNase IIIb domain.^1 2^

Missense mutations occurring in exons encoding the DICER1 RNase IIIb domain, either involving or adjacent to catalytically active metal‐ion binding residues including p.E1705, p.D1709, p.G1809, p.D1810, p.E1813 and p.D1713 are recognised as cancer hotspot loci. Alterations of these RNase IIIb residues cause neomorphic alleles, reported to be functionally equivalent with respect to miRNA biogenesis.^2^ These alterations interfere with the canonical processing of miRNA precursors, resulting in a relative excess of 3p-miRNA and a depletion of 5p-miRNA.^3^ It has recently been revealed through evolutionary and structural coupling analyses that RNase IIIa-S1344 site is in close proximity to the active cleft of RNase IIIb domain and that a mutation in RNase IIIa-S1344 exhibit the same pattern of 5p-miRNA loss as that resulting from RNase IIIb hotspot mutations.^4^ In 2014, Klein *et al* proposed a new *DICER1* related syndrome with the acronym GLOW (*G*lobal developmental delay, *L*ung cysts, *O*vergrowth, *W*ilms tumour), describing two children with mosaic missense hotspot mutations in *DICER1* affecting the RNase IIIb domain. A highly penetrant and severe phenotype has been shown in patients with mosaic^5 6^ or germline^7^ RNase IIIb mutations, compared with patients with classic DICER1 syndrome caused by germline LOF pathogenic variants.

Here, we show evidence that the germline c.4031C>T (p.S1344L) mutation in *DICER1* causes a severe subtype of DICER1 syndrome with intellectual disability (ID), macrocephaly, extensive bilateral lung cysts, early onset of Wilms tumour and well-differentiated fetal lung adenocarcinoma—a clinical spectrum similar to, but distinct from, the phenotype reported in two patients with GLOW syndrome with postzygotic hotspot mutations in exons encoding the RNase IIIb domain.

## Materials and methods

### Methods

Genomic DNA was extracted from peripheral blood, skin, saliva, oral mucosa, fresh tumour tissue and formalin-fixed, paraffin-embedded tumour tissue using standard protocols. DNA from both parents was extracted from peripheral blood.

### Sequencing and bioinformatic analysis

Standard 30x whole genome sequencing (WGS), exome sequencing (ES) and bioinformatic analysis were performed at Clinical Genomics, SciLifeLab, Stockholm, using the Illumina HiSeq X Ten platform. Single nucleotide variants were called using Mutation Identification Pipeline. Variants were filtered, removing variants with a frequency over 1% in the general population.^8^ A constructed gene panel based on HPO-terms (Macrocephaly HP:0000256, Polydactyly HP:0010442, Nephroblastoma HP:0002667) consisting of in total 476 genes were analysed regarding variants affecting coding regions or splicing (online supplemental table 1). The clinically relevant sequence variant was verified in the patient using Sanger sequencing. Carrier testing of the parents was performed using Sanger sequencing.

10.1136/jmedgenet-2020-107385.supp1 Supplementary data

### Digital PCR

Genomic DNA extracted from blood, skin, saliva and oral mucosa were amplified using 1X QuantStudio 3D Digital PCR Master mix and commercially available TaqMan assay for *DICER1* c.4031C>T mutation (Applied Biosystems, California, USA). DNA was quantified using Qubit fluorometry (Thermo Fisher Scientific, Massachusetts, USA). Then 14.6 μL of PCR reaction mixes were loaded into QS3D Digital 20K V2 chips (Applied Biosystems). Protocol was followed as previously described.^9^ Digital PCR data were analysed using PoissonPlus algorithm (V.4.4.10) with a 95% CI and a desired precision of 10% by QuantStudio 3D AnalysisSuite (V.3.1.2-PRC-build-03).

## Results

### Clinical description

**Figure 1:**
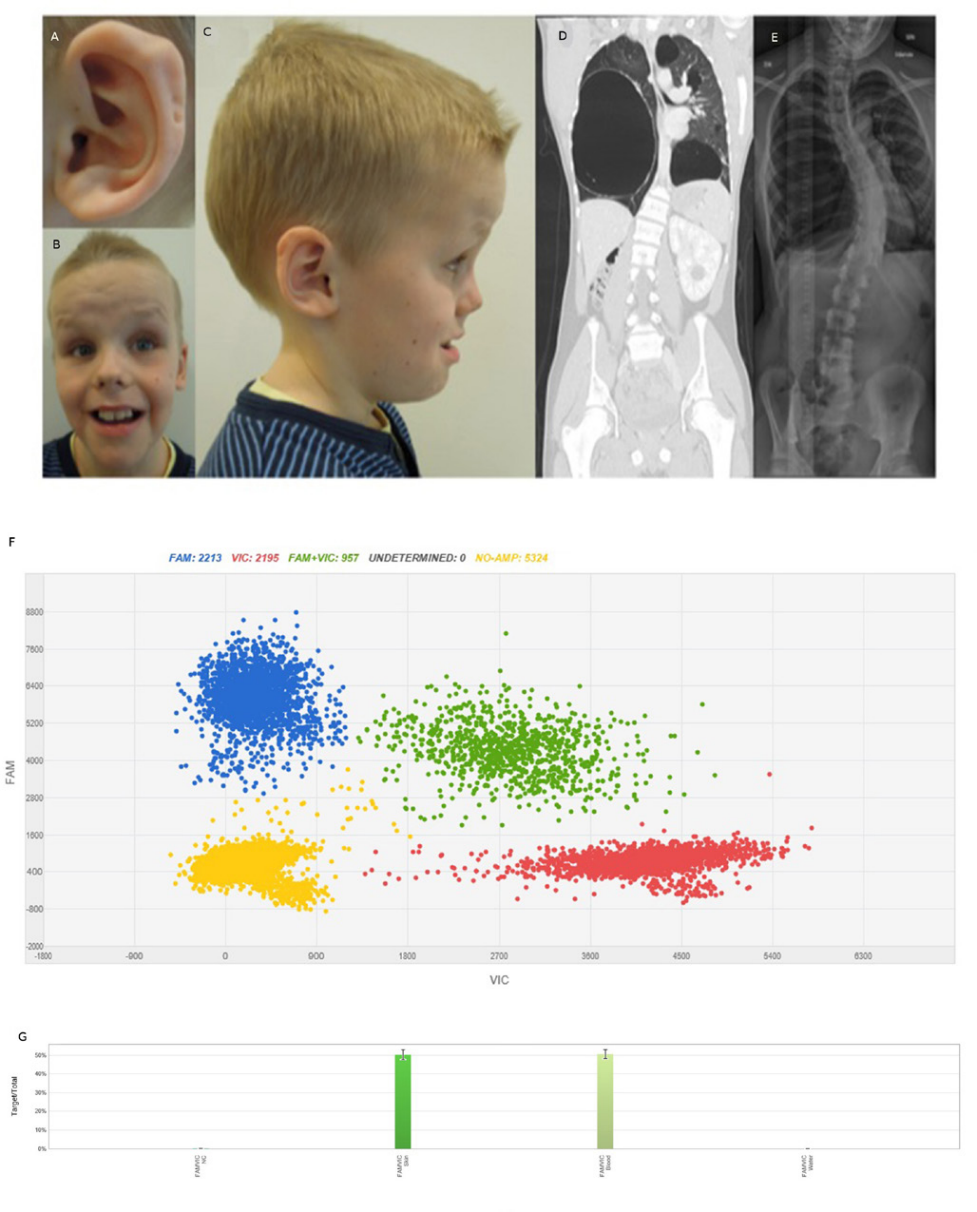
Patient characteristics. Photographs of the patient at 10.5 years of age showing (A) posterior ear pits of left helix, (B) a high, broad, furrowed forehead, mild hypertelorism, short, upturned nose, facial nevi, large mouth and teeth, (C) profile portrait of the patient showing macrocephaly and posterior ear pits of the right helix. (D) CT scan of the patient at 16 years of age showing bilateral large cysts and a cystic nephroma in the remaining enlarged kidney. (E) Chest X-ray of the patient at 16 years of age showing left convex thoracic scoliosis. (F) dPCR results of DNA from skin biopsy from the patient. Blue cluster represents amplification of the target region—the mutant allele c.4031C>T. Red signals represent the internal reference control and green signals both mutant and reference alleles. Yellow cluster represents the wells where no amplification signal was detected. (G) Mutant frequency comparison. X-axis and Y-axis show the intensities of signals VIC and FAM channels. Columns display the distribution of 50% of the c.4031C>T variant in DNA from skin and blood from the patient. Left column—skin, right column—peripheral blood.

The patient is the first child from healthy, non-consanguineous parents with unremarkable family history. He was born at gestational week 39 after a difficult delivery due to macrocephaly (Apgar 1-3-7). The birth weight was 3752 g, length 53 cm and head circumference 42 cm (>99th percentile). Clinical findings at birth included two blood vessels in the umbilical cord, undescended testis, inguinal hernia, postaxial polydactyly, ear pits and rocker bottom feet. Fontanel closure was late and he had difficulties breast feeding. At 15 months of age, he was diagnosed with a right-sided Wilms tumour (classic triphasic nephroblastoma with all three classic histological elements). Abdominal ultrasound also revealed a cyst of benign appearance in his left kidney. A lung scan showed multiple large cysts and a suspected Wilms tumour metastasis. A lobectomy of the middle lobe was performed to remove the tumour and three cysts. Owing to suspicion of additional metastasis, another thoracotomy was performed and two cysts in the right lower lobe were removed. The five removed lung cysts were diagnosed as benign fibrotic emphysematous alterations with bullous, cystic character. The pathologic-anatomic diagnosis of the lesion in the middle lobe was initially Wilms tumour metastasis, but following the identification of the pathogenic germline *DICER1* variant, pathology review determined that the correct diagnosis was well-differentiated fetal adenocarcinoma. Re-examination of radiographic findings, also after the DICER1 syndrome diagnosis, revealed a suspicion of a multiloculated cystic nephroma in the remaining kidney. The patient received postoperative chemotherapy and has been tumor-free since 2006. He had a late psychomotor development, started to walk at 3 years of age and spoke full sentences at 5 years. MRI of the brain was performed at 12 years of age and revealed signs of white matter reduction including a thin corpus callosum. At 18 years of age, he has normal length and weight, and a pronounced macrocephaly (>99th percentile). Dysmorphic features include a broad and furrowed forehead, wide mouth, tooth anomalies, doughy, soft skin and multiple nevi. Furthermore, he has ID, autism, behavioural problems and has required surgical treatment due to a left-convex thoracic scoliosis (figure 1, table 1).

**Table 1:** All known patients with germline or mosaic RNase III domain hotspot mutations

DICER1 syndrome+phenotype	Klein *et al* 5 Pat 1	Klein *et al*. Pat 2^5^	Pontén *et al (the present study)*r	Carlens *et al* 10	de Kock *et al*. 6 Pat 1 (Brenneman *et al* 11 Pat 102)	de Kock *et al* 6 Pat 2 (Brenneman *et al* 11 Pat 105)	de *Kock et al* 6 Pat 3	de Kock *et al* 6 Pat 4 Brenneman *et al* 11 Pat 120)	de Kock *et al* 7 Pat 12	Brenneman *et al* 11 Pat 101	Brenneman *et al* 11 Pat 103	Brenneman *et al*.^11^ Pat 104	Brenneman *et al* 11 Pat 123
Patient age and sex (, if reported)	5 y M	14 m M	18 y M	6 y F	10.9 y M	17.2 y F	6.8 y F	4.1 y M	21 m M	9 y	14 y	13 y F	8 y
*DICER1* RNase III hotspot mutation: domain/germline or mosaic	c.5138A>T p.D1713V RNase IIIb domain mosaic	c.5125G>T p.D1709Y RNase IIIb domain mosaic	c.4031C>T p.S1344L RNase IIIa domain germline	c.4031C>T p.S1344L RNase IIIa domain germline	c.5125G>A p.D1709N RNase IIIb domain mosaic	c.5437G>C p.E1813Q RNase IIIb domain mosaic	c.5439G>C p.E1813D RNase IIIb domain mosaic	c.5425G>A p.G1809R RNase IIIb domain mosaic	c.5125G>C p.D1709H RNase IIIb domain germline	c.5126A>G; p.D1709G RNase IIIb domain mosaic	c.5125G>A; p.D1709N RNase IIIb domain mosaic	c.5428G>T; p.D1810Y RNase IIIb domain mosaic	c.5113G>A; p.E1705K RNase IIIb domain mosaic
*DICER1* RNase III hotspot mutation: tissue distribution and VAF	Blood: (21%) Normal kidney: (35%) Wilms tumour:(37%)	Blood: (28%) Normal kidney:(35%) Wilms tumour:(47%)	Blood: (50%) Skin: (50%) Buccal cells: (50%) Saliva: (50%) Kidney tumour: (87%) Lung tumour: (90%)	Blood: (heterozygous)	Blood: (4.6%) Skin: (4.23%–7.13%) Saliva: (2.78%) Normal brain: (11.52%) Lymph node: (0%) NCMH: (17.72%) Type I PPB: (34.24%) Type II PPB: (43.84%) Brain PPB metastasis: (14.97%–51%)	Blood: (0.04%) Urine: (0.4%–0.66%) Saliva: (0.25%–0.27%) Hair: (0.24%) Lung cysts: (0%) Follicular thyroid ca:(66.6%–74.53%) SLCT (right): (76.8%–87.33%) SLCT (left): (31.8%–44.48%) NCMH: (29.3%–30.55%)	Blood: (0–0.04%) Normal right kidney #1: (3.26%–4.75%) Normal right kidney #2: (12.86%–14.55%) CN left kidney: (7.62%) Maldevelopmental lesion right kidney: (56.24%) Juvenile polyps: (38.9%) NCMH: (27.73%)	Blood: (0%–0.2%) Hair: (0.02%) Saliva: (0%–0.04%) Reactive lung (left): (0.35%–1%) Reactive lung (right): (6.93%–7.08%) Type II PPB: (37%–37.84%)	Blood: (heterozygous) Pituitary blastoma: (NR)	Blood: (NR) Normal lymph node: (15.2%)	Blood: (0.28%) CN: (14.9%) Type Ir PPB: (16.2%) Small intestine, polyps: (17.5%)	Blood: (0.21%) Normal Fallopian tube: (7.19%) Type Ir PPB: (27.8%) CN: (29.2%) SLCT (right): (34.2%) SLCT (left): (92.4%)	Blood: (ND) Normal ureter: (13%) Type I PPB:(24%) CN: (35%)
*DICER1* LOF mutation: tissue distribution/(allele frequency)	Blood: (ND) Normal kidney: (ND) Wilms tumour: c.1304C>A, p.P453H and c.5692A>G p.R1898G (variable)	Blood: (ND) Normal kidney: (ND) Wilms tumour: (ND)	Blood: (ND) Kidney tumour: 0.61 Mb deletion (approx. 50%)	Blood: (ND)	Blood: (ND) PPB metastasis in brain: (allele loss)	Blood: (ND) NCMH: (ND) Thyroid ca: (allele loss) SLCT (right): (allele loss) SLCT (left): c.4626-4626delG; p.Q1542Hfs*18: (21.7%–44.48%) NCMH: c.4458_4458delA p.K1486Nfs*4 (NR)	NCMH: c.4651-4652insTGCT; p.E1551Vfs*7 (NR)	Blood: (ND) Reactive lung: (ND) Type II PPB: c.1966C>T; p.R656* (NR)	Blood: (ND) Pituitary blastoma: (allele loss)	Blood: (ND) Normal Lymph node: (ND)	Blood: (ND) CN: c.1129G>A; p.V377I(3.1%) Type Ir PPB: c.1200G>A; p.W400* (4.1%) Small intestine polyp: c.96G>A; p.W32* (3%)	Blood: (ND) Normal Fallopian tube: (ND) Type Ir PPB: (ND) CN: c.1711delT; p.S571Vfs*16 (21.8%) SLCT (right): c.1775delA; p.K592Mfs*15 (36.2%) SLCT (left): (allele loss)	Blood: (ND) Normal ureter: (ND) Type I PPB: (allele loss) CN: (allele loss)
Intellectual impairment	DD	DD	ID	ID	ND	ND	ND	ND	ND	NR	NR	NR	NR
Autism	Yes	NR	Yes	Yes	NR	NR	NR	NR	NR	NR	NR	NR	NR
Hypotonia	Yes	Yes	Yes	Yes	NR	NR	NR	NR	NR	NR	NR	NR	NR
Overgrowth	Macrocephaly/Global	Macrocephaly/Global	Macrocephaly	Macrocephaly	ND	ND	ND	ND	ND	NR	NR	NR	NR
Birth weight	4904 g (>99th percentile)	2920 g (18th percentile)	3752 g (79th percentile)	4430 g (>99th percentile)	NR	NR	NR	NR	NR	NR	NR	NR	NR
Birth length	NR	NR	53 cm (95th percentile)	56 cm (>99th percentile)	NR	NR	NR	NR	NR	NR	NR	NR	NR
OFC at birth	NR	NR	42 cm (>99th percentile)	42 cm (>99th percentile)	NR	NR	NR	NR	NR	NR	NR	NR	NR
Growth parameters: weight (w)/length (L)/head size (OFC)	28 m: W 15.5 kg (91.9th percentile) L 95 cm (95.5th percentile) OFC 55 cm (>99th percentile)	14 m: W 13.5 kg (>99th percentile) L 81 cm (95.6th percentile) OFC 53 cm (>99th percentile)	18 y: W normal L normal OFC 62 cm (>99th percentile)	6 y: W 15.8 kg (3.6th percentile) L 112 cm (27.4th percentile)	NR	NR	NR	NR	NR	NR	NR	NR	NR
Nephromegaly	Yes	Yes	NR	No	NR	NR	NR	NR	NR	NR	NR	NR	NR
Renal cysts	Multiple small cysts	Multiloculated cystic mass (left)	CN at 8 y	NR	NR	Hamartomatous bilat renal cysts at 1 y 1 m	CN at 1 y	NR	Yes	CN at 1 y 3 m	CN at 1 y	CN at 1 y	CN at 1 y 6 m
Lung cysts/PPB	Lung cysts/PPB	Lung cysts/PPB	Type I PPB	Lung cysts at 11 m	Type I PPB Type II PPB	Benign multifocal bilat lung cysts at 1 y 1 m	Type I PPB (right) Lung cysts (left)	Lung cysts (right) Type I PPB (left) at 11 m, Type II PPB at 4 y	Type II PPB	PPB at 1 y 3 m	Type Ir PPB at 11 m	Type Ir PPB at 5 m	Type I PPB at 1 y 8 m
Hypertelorism	Yes	(Yes)	(Yes)	Yes	NR	NR	NR	NR	NR	NR	NR	NR	NR
Prominent forehead	Yes	Yes	Yes	Yes	NR	NR	NR	NR	NR	NR	NR	NR	NR
Anteverted nares	Yes	NR	Yes	NR	NR	NR	NR	NR	NR	NR	NR	NR	NR
Flat nasal bridge	Yes	Yes	Yes	NR	NR	NR	NR	NR	NR	NR	NR	NR	NR
Micrognathia	(Yes)	NR	(Yes)	NR	NR	NR	NR	NR	NR	NR	NR	NR	NR
Macrosomia	NR	NR	Yes	Yes	NR	NR	NR	NR	NR	NR	NR	NR	NR
Pits	Sacral dimple+dimples on sides of ankles	Ear pit (left)	Ear pits, posterior helix (left)	NR	NR	NR	NR	NR	NR	NR	NR	NR	NR
Skin findings	Doughy hands Fat pads on feet Pronounced plantar creases	NR	Soft skin Doughy soft hands Furrowed forehead Multiple nevi	NR	NR	NR	NR	NR	NR	NR	NR	NR	NR
Skeletal abnormalities	Pectus excavatum Kyphosis	NR	Scoliosis Polydactyly	Narrow thorax Increased intermammillary distance Prominent sternum	NR	NR	NR	NR	NR	NR	NR	NR	NR
Increased CSF space	Yes	Yes	Yes	Yes	NR	NR	NR	NR	NR	NR	NR	NR	NR
Fontanel	Large anterior fontanel	NR	Late fontanel closure	NR	NR	NR	NR	NR	NR	NR	NR	NR	NR
Hernia	Inguinal and umbilical	Inguinal	Inguinal	NR	NR	NR	NR	NR	NR	NR	NR	NR	NR
Brain imaging	Enlarged lateral and third ventricles	Mild volume loss	Mild volume loss Thin corpus callosum Cyst	Enlarged lateral ventricles Hydrocephalus	NR	NR	NR	NR	NR	NR	NR	NR	NR
Wilms tumour (age of Dx)	Bilateral 9 m/5 y	Bilateral 1 y 1 m/2 y 6 m	Bilateral 1 y 3 m/1 y 6 m	Unilateral 11 m	ND	ND	ND	ND	ND	ND	ND	ND	ND
Small intestine, polyps	NR	NR	NR	NR	Yes	NR	Hamartomatous juvenile intestinal polyps at 6 m	NR	NR	Yes	Yes	Yes	NR
Additional cysts or tumours	NR	NR	NR		NCMH at 8 y Testicular cyst at birth PPB brain metastasis	NCMH at 15 y Pineoblastoma at 7 y Follicular papillary thyroid carcinoma at 10 y Bilat SLCT at 13 y and 15 y Ciliary body medulloepithelioma at 17 y	NCMH at 6 y	NR	Pituitary blastoma at 8 m	Hodgkin lymphoma at 7 y	Pelvic sarcoma at 14 y	SLCT bilat at 5 y and 13 y Thyroid nodules at 7 y	NR
Other clinical findings	Respiratory distress	Pre-eclampsia in mother	Reduced fetal movement during pregnancy Retentio testis Rocker bottom feet Language impairment Large teeth Two blood vessels in umbilical cord Pulmonary embolism after scoliosis surgery	Low set ears Pulmonary infections Myopathic facies with ptosis and open mouth	Scrotal web at birth Enteritis cystica profunda at 9 m	Left ocular pre-phtisisical changes, vascular mass Recurrent retinal detachment	Renal medullary malformation with disorganised collecting system and dilated lymphatic vessels	NR	NR	NR	NR	NR	NR

CN, cystic nephroma; DD, developmental delay; ID, intellectual disability; LOF, loss of function; m, month; NCMH, nasal chondromesenchymal hamartoma; ND, none detected; NR, not reported; OFC, occipital frontal circumference; PPB, pleuropulmonary blastoma; SLCT, Sertoli-Leydig cell tumour; VAF, variant allele frequency; y, year.

### Genetic findings

WGS analysis after bioinformatic filtering revealed four sequence variants for clinical review (online supplemental table 2). The only clinically relevant variant was a heterozygous sequence variant in exon 21 of *DICER1;* NM_030621.4: c.4031C>T, (p.S1344L). The variant was verified and segregation analysis was performed using Sanger sequencing. Neither of the parents were carriers of this variant (data not shown). Digital PCR was performed on patient-DNA from buccal cells (not shown), saliva (not shown), blood and skin (figure 1), which showed a mutant allelic frequency of 50% in all samples, thus excluding mosaicism of the mutation. ES of DNA prepared from kidney and lung tumour tissue revealed that the germline heterozygous mutation c.4031C>T in *DICER1* was more abundant in the tumour tissue, 87% and 90%, respectively. In order to verify loss of heterozygosity (LOH) in the tumour, DNA from the fresh tissue (kidney tumour) was subjected to WGS. WGS data confirmed a 0.61 Mb deletion covering the whole *DICER1* gene (Chr14(Hg19): g.95247243_95858197del).

ES of the well-differentiated fetal lung adenocarcinoma revealed distinct tumour-only single nucleotide variants that distinguished it from the Wilms tumour and confirmed its separate nature.

## Discussion

We show that a heterozygous germline mutation c.4031C>T (p.S1344L) in the RNase IIIa domain of DICER1 is compatible with life, and causes a complex ‘DICER1 syndrome plus’ phenotype with extensive bilateral and multilobar lung cysts, PPB, cystic nephroma, Wilms tumour, well-differentiated fetal lung adenocarcinoma, ID, macrocephaly, ear pits and other physical anomalies. This phenotype closely resembles reported clinical findings of a patient with the identical germline p.S1344L pathogenic variant presented at a paediatric pneumology association meeting in Vienna.^10^ There are also close similarities to the phenotype described in two children with GLOW syndrome and mosaic hotspot mutations in the RNase IIIb domain,^5^ although the postnatal overgrowth was more pronounced in the individuals with GLOW syndrome. Thus, the phenotype of the widely expressed germline RNase IIIa-S1344L variant resembles the extreme end of the phenotypes associated with mosaic hotspot mutations in the sequence encoding the RNase IIIb domain of DICER1.^11^ This strongly supports previous somatic findings from integrated genetic analysis and in vitro cell experiments in cancer cells, where the RNase IIIa-S1344L mutation functionally perturbs RNase IIIb catalytic activity.^2 4^ The observation that only a minority of reported germline (n=1) or mosaic (n=10) RNase IIIb mutations are associated with the so-called GLOW phenotype (table 1), suggests that these mutations result in severe but highly pleiotropic phenotypes that cannot be predicted from the genotype.^2 5 6 11^ It will be important to determine whether, in contrast, RNase IIIa hotspot mutations invariably result in a severe ‘DICER1 syndrome plus’ phenotype.

In support of a potentially distinct functional consequence of RNase IIIa hotspot mutations, compared with RNase IIIb mutations, RNase IIIa catalytic site mutations are rare in cancers and RNase IIIa-S1344L is the only recurring cancer hotspot mutation that occurs in this domain. To date, p.S1344L mutations have been described in 15 tumours. Interestingly, the types of tumours which develop due to *DICER1* hotspot mutations are unevenly distributed among the targeted tissues. In the 15 tumours with p.S1344L in the RNase IIIa domain, there were 4 Wilms tumours, 4 malignant melanomas, 1 Merkel cell carcinoma, 3 endometroid carcinomas, 2 colorectal cancers and 1 cholangiocarcinoma.^10 12–15^ Tumours bearing RNase IIIa-S1344L hotspot mutations are most commonly malignant melanomas (4/15; 27%) in contrast to tumours bearing hotspot mutations in the RNase IIIb domain (2/318; 0.6%).^4 15^ This indicates that hotspot mutations in different RNase III domains of DICER1 cause tissue-specific susceptibilities to develop certain tumours.

This is the second well-differentiated fetal lung adenocarcinoma reported in DICER1 syndrome.^16–18^ Here, the diagnosis was made on the basis of glycogen-rich neoplastic glands and tubules resembling fetal lung tissue (at 10–15 weeks gestation). In contrast to biphasic PPB, the adjacent stroma is benign.

In addition to our patient, we identified 27 sequenced Wilms tumours with RNase IIIa or RNase IIIb domain hotspot mutations; in 17 of 28 (61%) there was no alteration on the other allele, while 11 of 28 (39%) had two mutations. One tumour with a germline RNase IIIa hotspot mutation did not undergo somatic testing (online supplemental table 3). Further studies are needed to determine if the sequenced Wilms tumours that lacked somatic alterations on the other allele could have copy neutral LOH or a deletion as described in our patient, or other genetic aberrations that escaped detection by the methods used.

10.1136/jmedgenet-2020-107385.supp2 Supplementary data

Non-tumour-related phenotypes occur in DICER1 syndrome; macrocephaly has recently been described in 28/67 (42%) of patients with the syndrome in a single study.^19^ General overgrowth is more pronounced in patients with GLOW syndrome,^5^ while patients with RNase IIIa-S1344L mutations only present with macrocephaly.^10^ Recently, Klein *et al* reported activation of the phosphatidylinositol 3-kinase (PI3K)/AKT/mammalian target of rapamycin (mTOR) pathway in genetically modified cells with hotspot mutations in the RNase IIIb domain associated with GLOW syndrome.^20^ It is noteworthy that some patients with mutations in the PI3K/AKT/mTOR pathway have overlapping symptoms with our patient, such as polydactyly in patients with mutations in *AKT3* or *PIK3CA*, and multiple nevi, macrocephaly and ID seen in *PTEN* and *PIK3CA*-related disorders. Posterior helical pits are rare and have previously been described in Beckwith-Wiedemann syndrome, Simpson-Golabi-Behmel syndrome and GLOW syndrome, all of which are cancer predisposition syndromes with a risk of developing Wilms tumour. In patients with Beckwith-Wiedemann syndrome and *CDKN1C* mutations, an extended phenotype including ear pits and polydactyly has been described. To date, ID/developmental delay has been reported in at least nine patients with large deletions encompassing several genes including *DICER1*,^21–27^ and in three patients with RNase IIIb or RNase IIIa-S1344L hotspot mutations.^5 10^

The highly penetrant and severe phenotypes described in patients with germline or mosaic RNase III domain mutations^5 6^ may be explained by the likelihood of a second somatic mutation stochastically occurring in any part of *DICER1* being greater than the reverse succession normally seen in DICER1 syndrome, in combination with tissue-specific neomorphic effects of the specific heterozygous RNase III domain mutations.^11^

In conclusion, germline RNase IIIa-S1344L pathogenic variants result in a "DICER1 syndrome plus" phenotype that in addition to classic features, also includes ID and ear pits. The conferred phenotype is similar, but not identical to that of early post zygotic DICER1 RNase IIIb hotspot mutations.
