# Supplementary material for: A complex DICER1 syndrome phenotype associated with a germline pathogenic variant affecting the RNase IIIa domain of DICER1
Source: J Med Genet. 2020 Nov 18;59(2):141–6. doi: 10.1136/jmedgenet-2020-107385 (PMC8788248; doi:10.1136/jmedgenet-2020-107385)
Supplement: Supplementary data [file jmedgenet-2020-107385supp001.pdf]

Supplementary Table 1; *List of analyzed genes*

HGNC symbol	HGNC ID	Description
ABCA12	14637	ATP binding cassette subfamily A member 12
ABCC9	60	ATP binding cassette subfamily C member 9
ACAN	319	aggrecan
ACOX1	119	acyl-CoA oxidase 1
ADK	257	adenosine kinase
AGGF1	24684	angiogenic factor with G-patch and FHA domains 1
AHI1	21575	Abelson helper integration site 1
AKT1	391	AKT serine/threonine kinase 1
AKT3	393	AKT serine/threonine kinase 3
ALX3	449	ALX homeobox 3
ALX4	450	ALX homeobox 4
AMER1	26837	APC membrane recruitment protein 1
ANKH	15492	ANKH inorganic pyrophosphate transport regulator
ANTXR2	21732	ANTXR cell adhesion molecule 2
AP1S2	560	adaptor related protein complex 1 subunit sigma 2
APC2	24036	APC2 WNT signaling pathway regulator
ARL13B	25419	ADP ribosylation factor like GTPase 13B
ARL6	13210	ADP ribosylation factor like GTPase 6
ARMC9	20730	armadillo repeat containing 9
ARSB	714	arylsulfatase B
ARVCF	728	ARVCF delta catenin family member
ASPA	756	aspartoacylase
ASXL2	23805	ASXL transcriptional regulator 2
B3GALNT2	28596	beta-1 3-N-acetylgalactosaminyltransferase 2
B3GALT6	17978	beta-1 3-galactosyltransferase 6
B3GLCT	20207	beta 3-glucosyltransferase
B4GALT1	924	beta-1 4-galactosyltransferase 1
B4GALT7	930	beta-1 4-galactosyltransferase 7
B4GAT1	15685	beta-1 4-glucuronyltransferase 1
B9D1	24123	B9 domain containing 1
B9D2	28636	B9 domain containing 2
BBIP1	28093	BBSome interacting protein 1
BBS1	966	Bardet-Biedl syndrome 1
BBS10	26291	Bardet-Biedl syndrome 10
BBS12	26648	Bardet-Biedl syndrome 12
BBS2	967	Bardet-Biedl syndrome 2
BBS4	969	Bardet-Biedl syndrome 4
BBS5	970	Bardet-Biedl syndrome 5
BBS7	18758	Bardet-Biedl syndrome 7
BBS9	30000	Bardet-Biedl syndrome 9
BGN	1044	biglycan
BHLHA9	35126	basic helix-loop-helix family member a9
BLM	1058	BLM RecQ like helicase
BMP2	1069	bone morphogenetic protein 2
BMP4	1071	bone morphogenetic protein 4
BMPR1B	1077	bone morphogenetic protein receptor type 1B
BRAF	1097	B-Raf proto-oncogene serine/threonine kinase
BRCA2	1101	BRCA2 DNA repair associated

BRWD3	17342	bromodomain and WD repeat domain containing 3
BUB1	1148	BUB1 mitotic checkpoint serine/threonine kinase
BUB1B	1149	BUB1 mitotic checkpoint serine/threonine kinase B
BUB3	1151	BUB3 mitotic checkpoint protein
C12orf57	29521	chromosome 12 open reading frame 57
C2CD3	24564	C2 calcium dependent domain containing 3
C8orf37	27232	chromosome 8 open reading frame 37
CAMTA1	18806	calmodulin binding transcription activator 1
CC2D2A	29253	coiled-coil and C2 domain containing 2A
CCDC22	28909	coiled-coil domain containing 22
CCND2	1583	cyclin D2
CD96	16892	CD96 molecule
CDC45	1739	cell division cycle 45
CDC73	16783	cell division cycle 73
CDCA7	14628	cell division cycle associated 7
CDKN1C	1786	cyclin dependent kinase inhibitor 1C
CEP104	24866	centrosomal protein 104
CEP120	26690	centrosomal protein 120
CEP164	29182	centrosomal protein 164
CEP290	29021	centrosomal protein 290
CEP41	12370	centrosomal protein 41
CEP55	1161	centrosomal protein 55
CEP57	30794	centrosomal protein 57
CFC1	18292	cripto FRL-1 cryptic family 1
CHD4	1919	chromodomain helicase DNA binding protein 4
CHD7	20626	chromodomain helicase DNA binding protein 7
CHN1	1943	chimerin 1
CHRNA7	1960	cholinergic receptor nicotinic alpha 7 subunit
CLCN7	2025	chloride voltage-gated channel 7
COG6	18621	component of oligomeric golgi complex 6
COL25A1	18603	collagen type XXV alpha 1 chain
COL2A1	2200	collagen type II alpha 1 chain
COL4A1	2202	collagen type IV alpha 1 chain
COLEC10	2220	collectin subfamily member 10
COMT	2228	catechol-O-methyltransferase
CPLX1	2309	complexin 1
CRB2	18688	crumbs cell polarity complex component 2
CREBBP	2348	CREB binding protein
CSPP1	26193	centrosome and spindle pole associated protein 1
CTBP1	2494	C-terminal binding protein 1
CUL4B	2555	cullin 4B
CWC27	10664	CWC27 spliceosome associated protein homolog
D2HGDH	28358	D-2-hydroxyglutarate dehydrogenase
DACT1	17748	dishevelled binding antagonist of beta catenin 1
DAG1	2666	dystroglycan 1
DDX59	25360	DEAD-box helicase 59
DEAF1	14677	DEAF1 transcription factor
DHCR24	2859	24-dehydrocholesterol reductase
DHCR7	2860	7-dehydrocholesterol reductase
DICER1	17098	dicer 1 ribonuclease III
DIS3L2	28648	DIS3 like 3'-5' exoribonuclease 2

DLL3	2909	delta like canonical Notch ligand 3
DMRT3	13909	doublesex and mab-3 related transcription factor 3
DNMT3A	2978	DNA methyltransferase 3 alpha
DNMT3B	2979	DNA methyltransferase 3 beta
DOCK6	19189	dedicator of cytokinesis 6
DPYD	3012	dihydropyrimidine dehydrogenase
DVL1	3084	dishevelled segment polarity protein 1
DVL3	3087	dishevelled segment polarity protein 3
DYNC2H1	2962	dynein cytoplasmic 2 heavy chain 1
DYNC2LI1	24595	dynein cytoplasmic 2 light intermediate chain 1
EBP	3133	EBP cholestenol delta-isomerase
EED	3188	embryonic ectoderm development
EFNB1	3226	ephrin B1
EFTUD2	30858	elongation factor Tu GTP binding domain containing 2
EIF2B1	3257	eukaryotic translation initiation factor 2B subunit alpha
EIF2B2	3258	eukaryotic translation initiation factor 2B subunit beta
EIF2B3	3259	eukaryotic translation initiation factor 2B subunit gamma
EIF2B4	3260	eukaryotic translation initiation factor 2B subunit delta
EIF2B5	3261	eukaryotic translation initiation factor 2B subunit epsilon
EML1	3330	EMAP like 1
EP300	3373	E1A binding protein p300
ERF	3444	ETS2 repressor factor
ERMARD	21056	ER membrane associated RNA degradation
ETFA	3481	electron transfer flavoprotein subunit alpha
ETFB	3482	electron transfer flavoprotein subunit beta
ETFDH	3483	electron transfer flavoprotein dehydrogenase
EVC	3497	EvC ciliary complex subunit 1
EVC2	19747	EvC ciliary complex subunit 2
EXT2	3513	exostosin glycosyltransferase 2
EZH2	3527	enhancer of zeste 2 polycomb repressive complex 2 subunit
FAM111A	24725	family with sequence similarity 111 member A
FANCB	3583	FA complementation group B
FBLN1	3600	fibulin 1
FBN1	3603	fibrillin 1
FGF10	3666	fibroblast growth factor 10
FGFR1	3688	fibroblast growth factor receptor 1
FGFR2	3689	fibroblast growth factor receptor 2
FGFR3	3690	fibroblast growth factor receptor 3
FGFRL1	3693	fibroblast growth factor receptor like 1
FH	3700	fumarate hydratase
FIBP	3705	FGF1 intracellular binding protein
FKRP	17997	fukutin related protein
FKTN	3622	fukutin
FLI1	3749	Fli-1 proto-oncogene ETS transcription factor
FLII	3750	FLII actin remodeling protein
FLNA	3754	filamin A
FMR1	3775	fragile X mental retardation 1
FOXP1	3823	forkhead box P1
FOXRED1	26927	FAD dependent oxidoreductase domain containing 1
FZD2	4040	frizzled class receptor 2
GABRD	4084	gamma-aminobutyric acid type A receptor delta subunit

GATA4	4173	GATA binding protein 4
GATA6	4174	GATA binding protein 6
GCDH	4189	glutaryl-CoA dehydrogenase
GDF1	4214	growth differentiation factor 1
GDF5	4220	growth differentiation factor 5
GDF6	4221	growth differentiation factor 6
GFAP	4235	glial fibrillary acidic protein
GJA1	4274	gap junction protein alpha 1
GJB6	4288	gap junction protein beta 6
GLI1	4317	GLI family zinc finger 1
GLI2	4318	GLI family zinc finger 2
GLI3	4319	GLI family zinc finger 3
GNAI3	4387	G protein subunit alpha i3
GNAQ	4390	G protein subunit alpha q
GNAS	4392	GNAS complex locus
GP1BB	4440	glycoprotein Ib platelet subunit beta
GPC3	4451	glypican 3
GPC4	4452	glypican 4
GRIA3	4573	glutamate ionotropic receptor AMPA type subunit 3
GUSB	4696	glucuronidase beta
H19	4713	H19 imprinted maternally expressed transcript
HDAC4	14063	histone deacetylase 4
HDAC6	14064	histone deacetylase 6
HELLS	4861	helicase lymphoid specific
HEPACAM	26361	hepatic and glial cell adhesion molecule
HERC1	4867	HECT and RLD domain containing E3 ubiquitin protein ligase family member 1
HES7	15977	hes family bHLH transcription factor 7
HESX1	4877	HESX homeobox 1
HEXB	4879	hexosaminidase subunit beta
HIRA	4916	histone cell cycle regulator
HIST1H1E	4718	histone cluster 1 H1 family member e
HNRNPK	5044	heterogeneous nuclear ribonucleoprotein K
HOXA13	5102	homeobox A13
HOXD13	5136	homeobox D13
HRAS	5173	HRas proto-oncogene GTPase
HSD17B4	5213	hydroxysteroid 17-beta dehydrogenase 4
HUWE1	30892	HECT UBA and WWE domain containing 1 E3 ubiquitin protein ligase
HYLS1	26558	HYLS1 centriolar and ciliogenesis associated
ICK	21219	intestinal cell kinase
IDS	5389	iduronate 2-sulfatase
IDUA	5391	iduronidase alpha-L-
IFT140	29077	intraflagellar transport 140
IFT172	30391	intraflagellar transport 172
IFT27	18626	intraflagellar transport 27
IFT43	29669	intraflagellar transport 43
IFT52	15901	intraflagellar transport 52
IFT74	21424	intraflagellar transport 74
IFT80	29262	intraflagellar transport 80
IFT81	14313	intraflagellar transport 81
IGBP1	5461	immunoglobulin binding protein 1
IGF2	5466	insulin like growth factor 2

IHH	5956	Indian hedgehog signaling molecule
INPP5E	21474	inositol polyphosphate-5-phosphatase E
INPPL1	6080	inositol polyphosphate phosphatase like 1
INTU	29239	inturned planar cell polarity protein
IQSEC2	29059	IQ motif and Sec7 domain 2
ISPD	37276	isoprenoid synthase domain containing
ITCH	13890	itchy E3 ubiquitin protein ligase
JMJD1C	12313	jumonji domain containing 1C
KCNAB2	6229	potassium voltage-gated channel subfamily A regulatory beta subunit 2
KCNJ1	6255	potassium voltage-gated channel subfamily J member 1
KCNJ8	6269	potassium voltage-gated channel subfamily J member 8
KCNQ1	6294	potassium voltage-gated channel subfamily Q member 1
KCNQ1OT1	6295	KCNQ1 opposite strand/antisense transcript 1
KDM1A	29079	lysine demethylase 1A
KDM5C	11114	lysine demethylase 5C
KIAA0556	29068	KIAA0556
KIAA0586	19960	KIAA0586
KIAA0753	29110	KIAA0753
KIF22	6391	kinesin family member 22
KIF7	30497	kinesin family member 7
KLLN	37212	killin p53 regulated DNA replication inhibitor
KPTN	6404	kaptin actin binding protein
KRAS	6407	KRAS proto-oncogene GTPase
L1CAM	6470	L1 cell adhesion molecule
L2HGDH	20499	L-2-hydroxyglutarate dehydrogenase
LAMB1	6486	laminin subunit beta 1
LARGE1	6511	LARGE xylosyl- and glucuronyltransferase 1
LBR	6518	lamin B receptor
LETM1	6556	leucine zipper and EF-hand containing transmembrane protein 1
LFNG	6560	LFNG O-fucosylpeptide 3-beta-N-acetylglucosaminyltransferase
LHX4	21734	LIM homeobox 4
LMBR1	13243	limb development membrane protein 1
LRP2	6694	LDL receptor related protein 2
LRP4	6696	LDL receptor related protein 4
LRP5	6697	LDL receptor related protein 5
LZTFL1	6741	leucine zipper transcription factor like 1
MAB21L2	6758	mab-21 like 2
MAFB	6408	MAF bZIP transcription factor B
MAN2B1	6826	mannosidase alpha class 2B member 1
MAP2K1	6840	mitogen-activated protein kinase kinase 1
MAP2K2	6842	mitogen-activated protein kinase kinase 2
MAP3K1	6848	mitogen-activated protein kinase kinase kinase 1
MAPK10	6872	mitogen-activated protein kinase 10
MBTPS2	15455	membrane bound transcription factor peptidase site 2
MECP2	6990	methyl-CpG binding protein 2
MED12	11957	mediator complex subunit 12
MEGF8	3233	multiple EGF like domains 8
MESP2	29659	mesoderm posterior bHLH transcription factor 2
MGAT2	7045	mannosyl (alpha-1 6-)-glycoprotein beta-1 2-N-acetylglucosaminyltransferase
MITF	7105	melanocyte inducing transcription factor
MKKS	7108	McKusick-Kaufman syndrome

MKS1	7121	Meckel syndrome type 1
MLC1	17082	megalencephalic leukoencephalopathy with subcortical cysts 1
MOCS1	7190	molybdenum cofactor synthesis 1
MOCS2	7193	molybdenum cofactor synthesis 2
MPDZ	7208	multiple PDZ domain crumbs cell polarity complex component
MSX2	7392	msh homeobox 2
MTM1	7448	myotubularin 1
MTOR	3942	mechanistic target of rapamycin kinase
MYH8	7578	myosin heavy chain 8
NDUFA1	7683	NADH:ubiquinone oxidoreductase subunit A1
NDUFA11	20371	NADH:ubiquinone oxidoreductase subunit A11
NDUFAF1	18828	NADH:ubiquinone oxidoreductase complex assembly factor 1
NDUFAF2	28086	NADH:ubiquinone oxidoreductase complex assembly factor 2
NDUFAF3	29918	NADH:ubiquinone oxidoreductase complex assembly factor 3
NDUFAF4	21034	NADH:ubiquinone oxidoreductase complex assembly factor 4
NDUFAF5	15899	NADH:ubiquinone oxidoreductase complex assembly factor 5
NDUFB3	7698	NADH:ubiquinone oxidoreductase subunit B3
NDUFB9	7704	NADH:ubiquinone oxidoreductase subunit B9
NDUFS1	7707	NADH:ubiquinone oxidoreductase core subunit S1
NDUFS2	7708	NADH:ubiquinone oxidoreductase core subunit S2
NDUFS3	7710	NADH:ubiquinone oxidoreductase core subunit S3
NDUFS4	7711	NADH:ubiquinone oxidoreductase subunit S4
NDUFS6	7713	NADH:ubiquinone oxidoreductase subunit S6
NDUFV1	7716	NADH:ubiquinone oxidoreductase core subunit V1
NDUFV2	7717	NADH:ubiquinone oxidoreductase core subunit V2
NEK1	7744	NIMA related kinase 1
NEK9	18591	NIMA related kinase 9
NELFA	12768	negative elongation factor complex member A
NF1	7765	neurofibromin 1
NFIA	7784	nuclear factor I A
NFIX	7788	nuclear factor I X
NKX2-5	2488	NK2 homeobox 5
NKX2-6	32940	NK2 homeobox 6
NKX3-2	951	NK3 homeobox 2
NLRC4	16412	NLR family CARD domain containing 4
NLRP3	16400	NLR family pyrin domain containing 3
NOTCH2	7882	notch 2
NPHP1	7905	nephrocystin 1
NPHP3	7907	nephrocystin 3
NR0B1	7960	nuclear receptor subfamily 0 group B member 1
NR5A1	7983	nuclear receptor subfamily 5 group A member 1
NRAS	7989	NRAS proto-oncogene GTPase
NSD1	14234	nuclear receptor binding SET domain protein 1
NSD2	12766	nuclear receptor binding SET domain protein 2
NUBPL	20278	nucleotide binding protein like
NXN	18008	nucleoredoxin
OFD1	2567	OFD1 centriole and centriolar satellite protein
OPHN1	8148	oligophrenin 1
OTX2	8522	orthodenticle homeobox 2
PALB2	26144	partner and localizer of BRCA2
PAX6	8620	paired box 6

PDE6D	8788	phosphodiesterase 6D
PDSS1	17759	decaprenyl diphosphate synthase subunit 1
PEX1	8850	peroxisomal biogenesis factor 1
PEX10	8851	peroxisomal biogenesis factor 10
PEX11B	8853	peroxisomal biogenesis factor 11 beta
PEX12	8854	peroxisomal biogenesis factor 12
PEX13	8855	peroxisomal biogenesis factor 13
PEX14	8856	peroxisomal biogenesis factor 14
PEX16	8857	peroxisomal biogenesis factor 16
PEX19	9713	peroxisomal biogenesis factor 19
PEX2	9717	peroxisomal biogenesis factor 2
PEX26	22965	peroxisomal biogenesis factor 26
PEX3	8858	peroxisomal biogenesis factor 3
PEX5	9719	peroxisomal biogenesis factor 5
PEX6	8859	peroxisomal biogenesis factor 6
PHF21A	24156	PHD finger protein 21A
PHF6	18145	PHD finger protein 6
PHF8	20672	PHD finger protein 8
PIBF1	23352	progesterone immunomodulatory binding factor 1
PIGA	8957	phosphatidylinositol glycan anchor biosynthesis class A
PIGN	8967	phosphatidylinositol glycan anchor biosynthesis class N
PIGT	14938	phosphatidylinositol glycan anchor biosynthesis class T
PIK3CA	8975	phosphatidylinositol-4 5-bisphosphate 3-kinase catalytic subunit alpha
PIK3R2	8980	phosphoinositide-3-kinase regulatory subunit 2
PITX1	9004	paired like homeodomain 1
PLCB4	9059	phospholipase C beta 4
PLG	9071	plasminogen
PNPLA6	16268	patatin like phospholipase domain containing 6
POC1A	24488	POC1 centriolar protein A
POLE	9177	DNA polymerase epsilon catalytic subunit
POMGNT1	19139	protein O-linked mannose N-acetylglucosaminyltransferase 1 (beta 1 2-)
POMGNT2	25902	protein O-linked mannose N-acetylglucosaminyltransferase 2 (beta 1 4-)
POMK	26267	protein-O-mannose kinase
POMT1	9202	protein O-mannosyltransferase 1
POMT2	19743	protein O-mannosyltransferase 2
POP1	30129	POP1 homolog ribonuclease P/MRP subunit
PORCN	17652	porcupine O-acyltransferase
POU1F1	9210	POU class 1 homeobox 1
POU6F2	21694	POU class 6 homeobox 2
PPP2R5D	9312	protein phosphatase 2 regulatory subunit B'delta
PRDM16	14000	PR/SET domain 16
PROP1	9455	PROP paired-like homeobox 1
PTCH1	9585	patched 1
PTCH2	9586	patched 2
PTDSS1	9587	phosphatidylserine synthase 1
PTEN	9588	phosphatase and tensin homolog
PTHLH	9607	parathyroid hormone like hormone
RAB23	14263	RAB23 member RAS oncogene family
RAB39B	16499	RAB39B member RAS oncogene family
RAC1	9801	Rac family small GTPase 1
RAI1	9834	retinoic acid induced 1

RBM10	9896	RNA binding motif protein 10
RERE	9965	arginine-glutamic acid dipeptide repeats
REST	9966	RE1 silencing transcription factor
RIPPLY2	21390	ripply transcriptional repressor 2
RIT1	10023	Ras like without CAAX 1
RNF125	21150	ring finger protein 125
RNF135	21158	ring finger protein 135
ROR2	10257	receptor tyrosine kinase like orphan receptor 2
RPGRIP1	13436	RPGR interacting protein 1
RPGRIP1L	29168	RPGRIP1 like
RREB1	10449	ras responsive element binding protein 1
RUNX2	10472	runt related transcription factor 2
SALL1	10524	spalt like transcription factor 1
SALL4	15924	spalt like transcription factor 4
SC5D	10547	sterol-C5-desaturase
SDCCAG8	10671	serologically defined colon cancer antigen 8
SDHB	10681	succinate dehydrogenase complex iron sulfur subunit B
SDHC	10682	succinate dehydrogenase complex subunit C
SDHD	10683	succinate dehydrogenase complex subunit D
SEC23A	10701	Sec23 homolog A coat complex II component
SEC23B	10702	Sec23 homolog B coat complex II component
SEC24C	10705	SEC24 homolog C COPII coat complex component
SEC24D	10706	SEC24 homolog D COPII coat complex component
SEMA3E	10727	semaphorin 3E
SERPINH1	1546	serpin family H member 1
SETBP1	15573	SET binding protein 1
SETD2	18420	SET domain containing 2
SH2B1	30417	SH2B adaptor protein 1
SHANK3	14294	SH3 and multiple ankyrin repeat domains 3
SHH	10848	sonic hedgehog signaling molecule
SHOC2	15454	SHOC2 leucine rich repeat scaffold protein
SHPK	1492	sedoheptulokinase
SIM1	10882	SIM bHLH transcription factor 1
SKI	10896	SKI proto-oncogene
SLC25A1	10979	solute carrier family 25 member 1
SLC26A2	10994	solute carrier family 26 member 2
SLC29A3	23096	solute carrier family 29 member 3
SLC2A10	13444	solute carrier family 2 member 10
SLC35D1	20800	solute carrier family 35 member D1
SMARCB1	11103	SWI/SNF related matrix associated actin dependent regulator of chromatin subfamily b member 1
SMO	11119	smoothened frizzled class receptor
SMOC1	20318	SPARC related modular calcium binding 1
SNX10	14974	sorting nexin 10
SNX14	14977	sorting nexin 14
SOS1	11187	SOS Ras/Rac guanine nucleotide exchange factor 1
SOST	13771	sclerostin
SOX9	11204	SRY-box 9
SPINT2	11247	serine peptidase inhibitor Kunitz type 2
SPRED1	20249	sprouty related EVH1 domain containing 1
SRY	11311	sex determining region Y
STRADA	30172	STE20 related adaptor alpha

SUFU	16466	SUFU negative regulator of hedgehog signaling
SUMF1	20376	sulfatase modifying factor 1
SUZ12	17101	SUZ12 polycomb repressive complex 2 subunit
SYN1	11494	synapsin I
TBCK	28261	TBC1 domain containing kinase
TBX1	11592	T-box 1
TBX15	11594	T-box 15
TBX3	11602	T-box 3
TBX6	11605	T-box 6
TCF12	11623	transcription factor 12
TCIRG1	11647	T cell immune regulator 1 ATPase H+ transporting V0 subunit a3
TCTEX1D2	28482	Tctex1 domain containing 2
TCTN1	26113	tectonic family member 1
TCTN2	25774	tectonic family member 2
TCTN3	24519	tectonic family member 3
TFAP2A	11742	transcription factor AP-2 alpha
TFAP2B	11743	transcription factor AP-2 beta
TGFBR1	11772	transforming growth factor beta receptor 1
TGFBR2	11773	transforming growth factor beta receptor 2
THRA	11796	thyroid hormone receptor alpha
TIMMDC1	1321	translocase of inner mitochondrial membrane domain containing 1
TMCO1	18188	transmembrane and coiled-coil domains 1
TMEM107	28128	transmembrane protein 107
TMEM126B	30883	transmembrane protein 126B
TMEM138	26944	transmembrane protein 138
TMEM216	25018	transmembrane protein 216
TMEM231	37234	transmembrane protein 231
TMEM237	14432	transmembrane protein 237
TMEM67	28396	transmembrane protein 67
TNFRSF11A	11908	TNF receptor superfamily member 11a
TNFRSF11B	11909	TNF receptor superfamily member 11b
TNFSF11	11926	TNF superfamily member 11
TP53	11998	tumor protein p53
TRAF3IP1	17861	TRAF3 interacting protein 1
TRIM32	16380	tripartite motif containing 32
TRIM37	7523	tripartite motif containing 37
TRIP11	12305	thyroid hormone receptor interactor 11
TRIP13	12307	thyroid hormone receptor interactor 13
TTC21B	25660	tetratricopeptide repeat domain 21B
TTC8	20087	tetratricopeptide repeat domain 8
TWIST1	12428	twist family bHLH transcription factor 1
UBE2A	12472	ubiquitin conjugating enzyme E2 A
UBE3A	12496	ubiquitin protein ligase E3A
UFD1	12520	ubiquitin recognition factor in ER associated degradation 1
UPF3B	20439	UPF3B regulator of nonsense mediated mRNA decay
UQCC2	21237	ubiquinol-cytochrome c reductase complex assembly factor 2
USP9X	12632	ubiquitin specific peptidase 9 X-linked
VAMP7	11486	vesicle associated membrane protein 7
WASHC5	28984	WASH complex subunit 5
WDPCP	28027	WD repeat containing planar cell polarity effector
WDR19	18340	WD repeat domain 19

WDR34	28296	WD repeat domain 34
WDR35	29250	WD repeat domain 35
WDR60	21862	WD repeat domain 60
WNT5A	12784	Wnt family member 5A
WNT7A	12786	Wnt family member 7A
WT1	12796	Wilms tumor 1
WWOX	12799	WW domain containing oxidoreductase
XYLT1	15516	xylosyltransferase 1
YME1L1	12843	YME1 like 1 ATPase
ZBTB20	13503	zinc finger and BTB domain containing 20
ZBTB24	21143	zinc finger and BTB domain containing 24
ZBTB42	32550	zinc finger and BTB domain containing 42
ZDHHC9	18475	zinc finger DHHC-type containing 9
ZFPM2	16700	zinc finger protein FOG family member 2
ZIC1	12872	Zic family member 1
ZIC3	12874	Zic family member 3
ZNF141	12926	zinc finger protein 141
ZNF423	16762	zinc finger protein 423
ZNF469	23216	zinc finger protein 469
ZSWIM6	29316	zinc finger SWIM-type containing 6

Supplementary Table 2; *Sequence variants correlating to patient phenotype*

**HGNC symbol, sequence variant**
DICER1, NM_030621 (ENST00000393063) c.4031C>T p.Ser1344Leu, heterozygous
GLI2, NM_005270 (ENST00000361492) c.1760C>T p.Thr587Met, heterozygous
LRP5, NM_002335 (ENST00000294304) c.3552C>T p.=, heterozygous
LRP5, NM_002335 (ENST00000294304) c.4466C>T p.Thr1489Met, heterozygous
