# Supplementary material for: A complex DICER1 syndrome phenotype associated with a germline pathogenic variant affecting the RNase IIIa domain of DICER1
Source: J Med Genet. 2020 Nov 18;59(2):141–6. doi: 10.1136/jmedgenet-2020-107385 (PMC8788248; doi:10.1136/jmedgenet-2020-107385)
Supplement: Supplementary data [file jmedgenet-2020-107385supp002.pdf]

Supplementary Table 3.
WT bearing RNase IIIa/IIIb hotspot mutations

	PUBLICATION			WT PATIENT INFORMATION		GERMLINE/MOSAIC DICER1 VARIANT				WILMS TUMOR/SOMATIC DICER1 MUTATION				NUMBER OF DICER1 MUTATIONS
	Reference	*PMID*	*Year*	*Case ID, age, sex*	*Phenotype*	*Exon*	*DNA change*	*Protein change*	*Domain*	*Exon*	*DNA change*	*Protein change*	*Domain*	
1	Pontén *et al.*	this case	2020	Swedish patient; 15 months, male	WT, WFDLA, Type I PPB, CN, ID, Macrocephaly	21	c.4031C>T	p.S1344L	RNase IIIa		0.61 Mb deletion spanning *DICER1*			Two mutations
2	Carlens *et al.*	conference pamphlet	2018	German patient; 11 months, female	WT, ID, LC bilateral, Macrocephaly	21	c.4031C>T	p.S1344L	RNase IIIa		Not analyzed			Tumor status unknown
3	Foulkes *et al.*	24676357	2011	Fam4:III-2; 4 years, male	WT	8	c.912_919dup	p.R307Qfs*8	TRBPBD	21	c.4031C>T	p.S1344L	RNase IIIa	Two mutations
4	Gadd *et al.*	28825729	2017	PAJNPI; 2,6 years, male	WT					21 + 17	c.4031C>T + c.2771T>G	p.S1344L + p.L924*	RNase IIIa	Two mutations
5	Klein *et al.*	24676357	2014	Case 1; 9 months, male	WT, LC bilateral, DD, OGS (GLOW syndrome)	24	c.5138A>T (mosaic)	p.D1713V (mosaic)	RNase IIIb	8 + 27	c.1304C>A + c.5692A>G	p.P453H + p.R1898G	Helicase C-Terminal + dsRBD	Two mutations
6	Klein *et al.*	24676357	2014	Case 2; 18 months, male	WT, LC bilateral, DD, OGS (GLOW syndrome)	24	c.5125G>T (mosaic)	p.D1709Y (mosaic)	RNase IIIb		No other mutation			Monoallelic
7	Foulkes *et al.*	24676357	2011	Fam5:II-1; 2 years, male	WT	8	c.1306dup	p.S436Ffs*41	btwn TRBPBD and HELICc	25	c.5138A>C	p.D1713A	RNase IIIb	Two mutations
8	Foulkes *et al.*	24676357	2011	Fam3:IV-3; 5 years	WT, MNG	intron 13/junction exon 14	c.2117-1G>A	p.G706Afs*8	DUF	24	c.5429A>G	p.D1810G	RNase IIIb	Two mutations
9	Rakheja *et al.*	25190313	2014	CMCW11; 5,2 years, female	WT, ASK, Thyroid-FA, ERMS, SLCT bilateral	21	c.3307_3311del	p.I1102fsdel	btwn PAZ and RNase IIIa	25	c.5425G>A	p.G1809R	RNase IIIb	Two mutations
10	Wu *et al.*	23620094	2013	Case N	WT					25	c.5438A>G + c.5452G>A	p.E1813G + p.A1818T	exon 25 skip + RNase IIIb	Two mutations
11	Gadd *et al.*	28825729	2017	PAJNRH; 3,7 years + 4 years, female	WT bilateral?					27 + 24	c.5622C>G + c.5125G>A	p.Y1874* + p.D1709N	dsRBD + RNase IIIb	Two mutations
12	Gadd *et al.*	28825729	2017	PAJLVL; 5,4 years, female	WT					24 + 6	c.5138A>T + c.734+1T>C	p.D1713V + NMD	RNase IIIb	Two mutations
13	Gadd *et al.*	28825729	2017	PAKJJF; 3,2 years, female	WT					19 + 24	c.3008G>A + c.5125G>A	p.R1003Q + p.D1709N	PAZ + RNase IIIb	Two mutations
14	Walz *et al.*	25670082	2015	ValidationSetFHWT5	WT					24	c.5125G>	p.D1709N	RNase IIIb	Monoallelic
15	Walz *et al.*	25670082	2015	ValidationSetFHWT6	WT					24	c.5125G>	p.D1709N	RNase IIIb	Monoallelic
16	Gadd *et al.*	28825729	2017	PAJLXH; 8,3 years, female	WT					24	c.5125G>A	p.D1709N	RNase IIIb	Monoallelic
17	Gadd *et al.*	28825729	2017	PAJMWN; 2,1 years, female	WT					25	c.5125G>A	p.D1709N	RNase IIIb	Monoallelic
18	Walz *et al.*	25670082	2015	ValidationSetFHWT3	WT					24	c.5113G>	p.E1705K	RNase IIIb	Monoallelic
19	Walz *et al.*	25670082	2015	ValidationSetFHWT4	WT					24	c.5113G>	p.E1705K	RNase IIIb	Monoallelic

20	Gadd *et al.*	28825729	2017	PAJNBW; 2,6 years, female	WT		24	c.5113G>A	p.E1705K	RNase IIIb	Monoallelic
21	Gadd *et al.*	28825729	2017	PAJPAH; 3 years, female	WT		24	c.5113G>A	p.E1705K	RNase IIIb	Monoallelic
22	Gadd *et al.*	28825729	2017	PALEZT; 12 years, female	WT		24	c.5113G>A	p.E1705K	RNase IIIb	Monoallelic
23	Gadd *et al.*	28825729	2017	PAJLML; 3,9 years, male	WT		25	c.5428G>A	p.D1810N	RNase IIIb	Monoallelic
24	Gadd *et al.*	28825729	2017	PAJMVL; 2,7 years, male	WT		25	c.5428G>A	p.D1810N	RNase IIIb	Monoallelic
25	Torrezan *et al.*	24909261	2014	ACC13	WT		25	c.5428G>A	p.D1810N	RNase IIIb	Monoallelic
26	Walz *et al.*	25670082	2015	ValidationSetFHWT8	WT		25	c.5428G>	p.D1810N	RNase IIIb	Monoallelic
27	Walz *et al.*	25670082	2015	ValidationSetFHWT9	WT		25	c.5428G>	p.D1810N	RNase IIIb	Monoallelic
28	Walz *et al.*	25670082	2015	ValidationSetFHWT7	WT		25	c.5138A>	p.D1713V	RNase IIIb	Monoallelic
29	Rakheja *et al.*	25190313	2014	CMCW59; 3,5 years, male	WT		25	c.5426G>T	p.G1809V	RNase IIIb	Monoallelic

Abbreviations: ASK, anaplastic sarcoma of the kidney; CN, cystic nephroma; DD, developmental delay; ERMS, embryonal rhabdomyosarcoma; ID, intellectual disability; MNG, multinodular goitre; PPB, pleuropulmonary blastoma; OGS, overgrowth syndrome; LC, lung cysts; SLCT, Sertoli-Leydig cell tumor; Thyroid-FA, thyroid follicular adenoma; WT, Wilms tumor; WDFLA, well differentiated fetal lung carcinoma dsRBD, double stranded RNA binding domain; DUF, domain of unknown function, TRBPBD, TAR RNA binding protein binding domain.

Number of Cases	Description
4	p.S1344L
29	WT bearing a *DICER1* RNase IIIa/IIIb hotspot mutation*
11	WT bearing two *DICER1* alterations
17	WT bearing a single *DICER1* RNase IIIa/IIIb hotspot alteration
1	WT bearing germline *DICER1* RNase IIIa mutation; tumor status not known

**p.E1705, p.D1709, p.G1809, p.D1810, p.E1813, p.D1713, p.S1344L*
